# Home caregiving, early learning and the development of preschool-aged children in Vanuatu: A secondary analysis of 2023 Multiple Indicator Cluster Survey data

**DOI:** 10.1371/journal.pgph.0006325

**Published:** 2026-04-24

**Authors:** Sally Popplestone, Thach Tran, Yeji Baek, Shazna M. Buksh, Jane Fisher

**Affiliations:** 1 Global and Women’s Health, Public Health and Preventive Medicine, Monash University, Melbourne, Australia; 2 Health Economics and Policy Evaluation Research (HEPER), Centre for Medicine Use and Safety, Faculty of Pharmacy and Pharmaceutical Science, Monash University, Parkville, Australia; 3 School of Law and Social Science, The University of the South Pacific, Suva, Fiji; PLOS: Public Library of Science, UNITED STATES OF AMERICA

## Abstract

Optimal early childhood development (ECD) is foundational for lifelong learning, health, and wellbeing. As children spend most of their early years at home, this setting represents a modifiable environment for enhancing ECD. As yet, there is little ECD research in Vanuatu. The first aim was to determine the proportion of children aged 24–59 months who are developmentally on track and the distributions of home responsive caregiving, early learning and sociodemographic factors. The second was to describe associations between these factors and child development, and the third was to identify factors that moderate these associations. Data were drawn from the 2023 Vanuatu Multiple Indicator Cluster Survey, which included the Early Childhood Development Index 2030. Among 1154 eligible children, 68.2% were developmentally on track. Associations between ECD and 22 explanatory factors were tested using multiple linear regression. Adjusted analysis showed significantly better developmental scores among children with access to three or more books, two or more playthings, receiving three forms of positive parenting, experiencing adequate stimulation and responsive care from their mothers, fathers and nonparental caregivers. Higher development scores were also associated with older age, urban residence, the second highest wealth quintile, and living in the northeast Penama region. Findings difficult to interpret include that higher scores were recorded from children whose families justified domestic violence and those who experienced inadequate supervision. Null findings were reported for ten of the twenty-two factors. Moderation analyses explored how the associations between home factors and child development change depending on socio-demographic factors, with variations identified across sex, urbanicity and maternal education. For example, the associations between positive parenting and child development were stronger for girls than boys, urban than rural children, and children of mothers with lower versus higher education, suggesting positive parenting may play a more important role in supporting their development. Overall, these findings suggest key policy priorities, including improving access to books and toys, investing in parenting education to enhance home-based caregiving strategies and developmental support, and tailoring programs to regional and rural contexts.

## Introduction

Optimal early childhood development (ECD) provides the foundation for lifelong learning, health, and wellbeing [1]. Currently, an estimated 80.8 million children aged three to four years in 35 low and middle-income countries (LMICs) (32.9%) have low cognitive and/or social emotional development [2]. Low cognitive development is characterised by difficulties in following simple instructions and working independently, while low social emotional development includes problems with emotion regulation, aggression, attention, and peer interaction [2]. Lower child development is more commonly observed in resource-constrained settings, where poverty and deficient caregiving environments can have negative impacts on early childhood brain development [3–5]. Longitudinal data indicates that children from the poorest households, rural areas, and marginalised social groups consistently experience poorer nutritional status and learning outcomes [6].

During the critical early childhood window from conception to eight years of age, children experience rapid and foundational development across cognitive, physical, language, motor, and social-emotional domains [7]. Within this broader phase, the preschool period (aged two to five years) is considered an important life stage [8,9]. Building on developmental gains made in infancy, this period also provides an opportunity for physical and cognitive catch up growth, especially for children whose development may be faltering [10–14]. The preschool period is characterised by heightened neural maturation, rapid motor, language and social-emotional domain growth, and the expansion of self-regulation and executive functioning skills [15]. Development during this phase is often hindered by poverty and related risks, including poor nutrition, inadequate healthcare, neglect, limited caregiving, experiences of violence and restricted access to early learning [8].

The World Health Organization’s Nurturing Care Framework outlines the five dimensions necessary for optimal child development: good health, nutrition, safety, early learning, and responsive caregiving [7]. Responsive caregiving refers to a caring and understanding relationship between a caregiver and child, which includes anticipatory guidance for safety, education, and development, as well as responsive and sensitive interactions [7]. More broadly, nurturing care is supported by a range of social and structural environments including the home, early childhood services, schools, communities, and government policy. The quality of nurturing care that children experience in their immediate surroundings, such as in the home or child-care settings, most strongly influences their development [16].

Given children spend most of their early years at home, the home environment is a key focus of research due to its modifiable potential for improving early childhood development. Positive parenting practices, such as attentive and emotional engagement, and cognitive stimulation with a child, are associated with higher child development [17–24]. Home-based early learning activities such as reading, storytelling, playing, naming objects, and having books in the home are linked to higher developmental scores among pre-school aged children [25–31]. Despite its potential, an estimated 182 million children aged three and four years in LMICs (75%) currently lack minimally adequate nurturing care, across at least one component each of the five dimensions of nurturing care [32]. In particular, the experience of nurturing is lowest for the responsive caregiving and early learning dimensions, compared with the dimensions of health and nutrition [32].

Ecological and developmental theories, along with empirical studies, highlight the significant influence of the home environment on child development [2,33,34]. Prior evidence also emphasises that children’s development is shaped by multiple interacting layers of influence ranging from individual characteristics to broader social and structural conditions [1,3,33]. Because families’ ability to provide nurturing care depends on the socioeconomic and environmental context in which they live, a comprehensive set of predictors is required to capture the diverse factors that influence child development through both direct and indirect pathways. Guided by this perspective, the present study adopts a conceptual framework that organises potential determinants of child development across child, women and household characteristics, mother safety, security, and gender equality factors, and indicators of the home caregiving and early learning environment.

In the East Asia and Pacific region, research across LMICs highlights significant variation in child development, shaped by cultural, socio-economic, and policy differences, as well as disparities in parenting practices and access to early learning [35]. A multi-country secondary analysis including Vanuatu found that home learning activities, preschool attendance and duration were positively associated with child development [36]. However, the study did not report country-specific findings for Vanuatu and focused only on three and four year olds. Another regional study found that, in Vanuatu, parental engagement with three and four years old in social-emotional activities significantly moderated socioeconomic gradients in development but did not examine learning materials, supervision or discipline practices [30]. More recently, a Fiji study identified positive associations between child development elements of the home caregiving environment including having books in the home and positive parenting [31]. These findings reinforce the influence of the home environment on child development and highlight the need for country-specific data in Vanuatu.

Vanuatu has made progress in prioritising ECD through the adoption of its first multisectoral national policy for early childhood development 2023–2027 [37]. This policy builds on a regional collaboration through the 2017 Pasifika Call to Action on Early Childhood Development, championed by 15 Pacific countries including Vanuatu [38]. Yet, population-level evidence on how home-based early learning and responsive caregiving relate to child development remains limited. Addressing these evidence gaps is essential not only for advancing Vanuatu’s policy goals, but also for meeting international commitments to monitor and improve early childhood outcomes.

Ensuring optimal ECD, care and pre-primary education are global health priorities articulated in the United Nations Sustainable Development Goals (SDGs) target 4.2. Indicator 4.2.1 measures the proportion of children aged 24–59 months who are developmentally on track in health, learning, and psychosocial wellbeing, by sex [39]. The 2023 launch of UNICEF’s globally standardized Early Childhood Development Index 2030 (ECDI2030) provides a tool to measure developmental progress in this age group and to support national monitoring of SDG 4.2.1.

As ECDI2030 is embedded within UNICEF’s Multiple Indicator Cluster Surveys (MICS), it enables population-level assessment of child development across diverse country settings. While this global tool provides valuable cross-country comparability, some of its constructs may not fully capture the breadth of culturally normative caregiving practises in Pacific contexts. For example, survey measures such as book ownership, specific play activities, or the availability of toys may not capture all developmentally rich practises [40]. Structural factors including poverty, parental workload, and limited literacy can also influence the types of activities caregivers can engage in, without indicating a lack of interest or competence [1,41]. Recognising these limitations helps to prevent deficit framing. At the same time, country-level data remain essential for informing evidence-based policies that address the needs of Vanuatu’s preschool aged population, including two year olds who have previously been overlooked.

Our aims were to [1] determine the proportion of children aged 24–59 months who are developmentally on track and the distributions of home responsive caregiving, early learning and sociodemographic factors, [2] describe the associations between home responsive caregiving and learning indicators, sociodemographic factors, and the development of children aged 24–59 months in Vanuatu, and [3] identify factors that may moderate these associations.

## Methods

### Study design

This is a secondary analysis of quantitative data from the UNICEF Multiple Indicator Cluster Survey (MICS) conducted in Vanuatu in 2023.

### Setting

Vanuatu is a Y-shaped archipelago located in the Melanesian region of the Pacific Ocean, characterised by mountainous terrain and narrow coastal plains. It comprises six provinces: Torba, Sanma, Penama, Malampa, Shefa, and Tafea, with each encompassing a group of islands. Of the country’s 83 islands, 65 are inhabited by a population of approximately 320,000 [42]. Vanuatu has three official languages: Bislama, English, and French. Bislama is the most widely spoken, while English is the primary language used in education, government, and business [42].

Classified as a lower middle-income country by the World Bank, Vanuatu’s economy relies on agriculture, tourism, offshore financial services, and international aid. The country faces significant poverty with approximately 19% of the population living on less than three US dollars per day [43]. Vanuatu is also one of the most disaster-prone countries globally, exposed to frequent earthquakes, cyclones, and volcanic activity [44]. In 2023, Category 4 Tropical Cyclones Judy and Kevin struck just one month apart, affecting over 80% of the population [45]. These cyclones occurred only a few months prior to the Vanuatu MICS data collection [45].

Children under five years of age make up approximately 14% of the population, with 29.1% experiencing stunting. Early childhood education enrolment among three and four year olds is around 39% [45,46]. The Human Capital Index (HCI) measures the human capital a child can expect to attain by age 18. Vanuatu’s HCI is 0.455, meaning a child born in Vanuatu today is projected to be only 45.5% as productive as they could be with optimal health and education [47]. This score is below the regional East Asia and Pacific average of 59%.

Parenting practises and gender roles in Vanuatu are shaped by broader Melanesian social norms, where caregiving responsibilities are often gendered. Mothers typically undertake most daily childcare, early learning activities, and household labour, while fathers’ roles more commonly involve decision making, discipline, and resource provision [48]. Gender norms also shape attitudes towards family relationships and the use of physical discipline, with national surveys documenting relatively high acceptance of intimate partner violence in Vanuatu [49]. Gendered inequality across Pacific Island countries is shaped not only by high rates of intimate partner violence, but also limited representation of women in decision making, and restricted access to employment and economic opportunities, which may reinforce persistent inequities in the region [50].

### Sampling, participants, and procedures

#### Multiple indicator cluster survey in Vanuatu.

UNICEF’s MICS are quantitative household surveys conducted at the country level to inform policy, planning, and progress towards the SDGs [51]. The 2023 Vanuatu MICS was an interviewer administered face-to-face survey conducted by the Vanuatu Bureau of Statistics, with support from UNICEF’s Pacific Multi-Country Office, UNFPA’s Pacific sub-office staff, and UNICEF’s Global MICS team [45].

The survey in Vanuatu covered urban and rural areas, and the country’s six provinces. A two-stage sampling process was employed. First, 238 enumeration areas were systematically selected from the 2020 census using probability proportional to size. Although the 2022 Agricultural Census household list was initially intended for use, cyclone disruptions limited its availability to only 31 areas, necessitating reliance on the 2020 census list for the remaining 207. Second, within each of the selected enumeration areas, a systematic sample of 20 households were selected in the primary sampling units in Torba, Penama, and Malampa, and 24 households in the primary sampling units in Shefa and Tafea. To account for expected nonresponse in cyclone affected provinces, larger household samples were drawn in Shefa and Tafea. Ultimately, 5132 households were targeted, but one cluster in Torba was inaccessible due to weather, reducing the final sample to 5112 households.

The Vanuatu MICS data were collected using six questionnaires. The six questionnaires together gathered information from: the household head on household composition and living conditions; all women aged 15–49 on their individual characteristics and wellbeing; all men aged 15–49 (in every second household) on their individual characteristics and wellbeing; mothers or primary caregivers on the development of all children aged under five years; the mother or primary caregiver of one randomly selected child aged 5–15 in each selected household; and household water quality from selected households in each sample area. Our study included data drawn from the individual women’s questionnaire and the under-five questionnaire. Caregiver reported information captured in these surveys included answers to questions about child development, health, nutrition, child protection, responsive caregiving, and early learning. In each participating household, all women aged 15–49 completed the women’s questionnaire, and mothers or caretakers completed a questionnaire for every child aged under five years.

The MICS6 questionnaires were customised and translated from English into Bislama and French, and pre-tested in both urban and rural areas around Port Villa during April and May 2023. Computer assisted personal interviewing (CAPI) tablets were used to conduct the questionnaires. Teams were trained during a one month period. The CAPI pre-test results informed wording and translation modifications to the final questionnaires. The data were collected between July and October 2023 by ten teams, each comprising four interviewers, one driver, one measurer and one supervisor. Further details are found elsewhere [45].

#### Inclusion criteria.

Our study included data from mothers in the Vanuatu MICS who provided complete information on home-based early learning and responsive caregiving and child development for their children aged 24–59 months.

### Measurements

**Fig 1:**
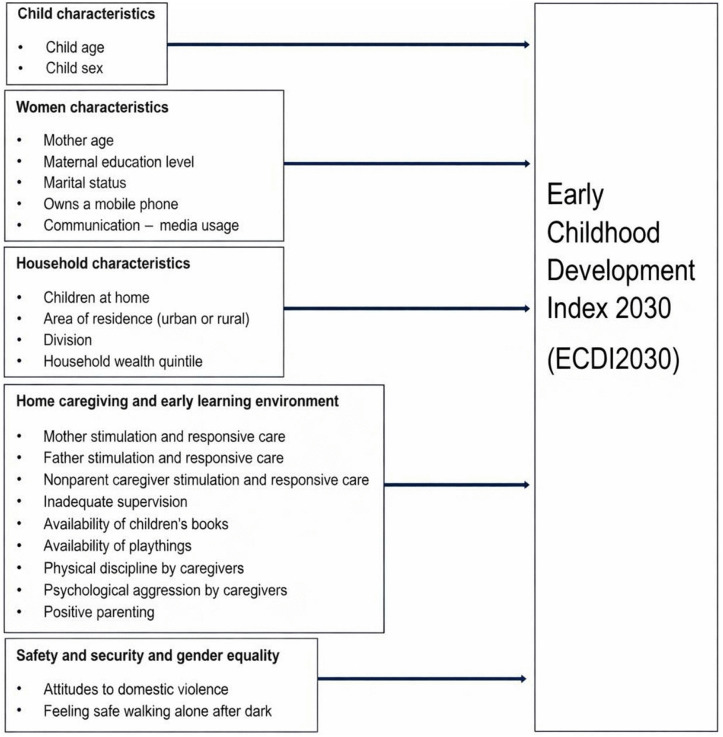
Conceptual framework of the study.

To reflect the multifaceted influences on child development, the conceptual framework of this study is structured around five areas: child characteristics, women’s characteristics, household characteristics, the home caregiving and early learning environment, and mother safety, security and gender equality (Fig 1). The selection of 22 factors reflects a bioecological understanding of early development, in which children’s outcomes are shaped by multiple, interacting layers of their environment [33]. The variables within this framework are a mix of known and exploratory factors. Variables such as maternal education, household wealth, and child age form the core of the model given their well-established associations with a ECD [19,24,30,52]. The framework also incorporates less commonly examined factors, for example, maternal mobile phone ownership, attitudes towards domestic violence, and perceived safety walking alone after dark. These represent exploratory predictors aimed at capturing the multidimensional nature of the home environment. Attitudes towards domestic violence serve as a proxy for gender norms. Perceived safety after dark signals broader community conditions and caregiver stress, which may indirectly shape the home environment. Maternal mobile phone ownership functions as an indicator of access to information, communication and social networks, which may also affect the home environment via maternal access to information and services.

#### Outcome.

The Early Childhood Development Index 2030 (ECDI2030) is a globally validated population level measure for children aged 24–59 months. The ECDI2030 has demonstrated construct, face and discriminant validity through associations with established child development measures, such as the International Learning and Early Development Assessment and Measuring Early Learning and Quality Outcomes, as well as the Early Childhood Development Assessment Scale – Direct Assessment as a direct comparator to its caregiver-reported items [53]. It demonstrates strong psychometric properties, including high internal consistency (reliability coefficients >0.83), robust item discrimination and difficulty across age groups, and cross cultural validity through cognitive testing and field trials in over 30 countries [54]. The ECDI2030 is a 20-item questionnaire that was introduced in 2023 and is now embedded as a routine child development module in MICS surveys.

Through interviews with mothers of children aged 24–59 months, the ECDI2030 questionnaire assesses children’s behaviour, skills, and knowledge across three domains: learning (11 questions), health (5 questions), and psychosocial well-being (4 questions). Examples of questions across the three domains: Learning: Can (*name*) say 10 or more words? Can (*name*) count 10 objects without mistakes?; Health – Can (*name*) walk on an uneven surface without falling?; Psychosocial well-being *–* Does (*name*) ask about familiar people other than parents when they are not present?

We assessed child development using a continuous ECDI2030 scale in the regression models to ensure precision and enhanced statistical power for interpreting the associations. We also calculated the ECDI2030 milestone achieved using the UNICEF–specified ECDI2030 scoring method to determine whether two, three and four year olds were considered developmentally ‘on track’. The indicator derived from the ECDI2030 is the percentage of children aged 24–59 months who have achieved a minimum number of milestones expected for their age group, classified as developmentally on track or not. The ECDI2030 scoring method calculates if a child meets the age-specific minimum milestones employing the following cut-off scores out of a possible total of 20 milestones: 24–29 months seven milestones; 30–35 months: nine milestones; 36–41 months: 11 milestones, 42–47 months: 13 milestones, and 48–59 months: 15 milestones [54].

#### Explanatory factors.

Explanatory factors were drawn from answers provided by mothers about questions related to home-based early learning and responsive caregiving. The explanatory factors included: receiving adequate early stimulation and responsive care by mother; receiving adequate early stimulation and responsive care by father; receiving adequate early stimulation and responsive care by someone other than mother or father; availability of children’s books; availability of playthings; inadequate supervision; violent physical discipline; psychological aggression; and positive parenting (Table 1). The coding and scoring of the home-based early learning and responsive caregiving variables followed the definitions and indicators set out in the MICS 2023 Vanuatu Survey Findings Report, except for the positive parenting variable [45]. For positive parenting, we created a study-specific binary variable distinguishing children experiencing zero to two forms of positive parenting from those reporting all three. This approach ensured that any evidence of positive parenting was captured, rather than comparing only none versus three.

**Table 1:** Home-based early learning and responsive caregiving constructs, measures, coding and scoring.

Construct	Measure	Coding and scoring
Receiving adequate early stimulation and responsive care	Engaging in four or more of the following six activities in the past three days: read books, tell stories, sing songs, take outside, play with, name/count.	A summary score was created by recoding and computing six questions each for the mother and father. This was further recoded into a binary variable to quantify adequate versus insufficient early stimulation and responsive care, defined by whether the child received four or more activities of early stimulation and responsive care.
Availability of children’s books	Three or more books at home.	Availability of children’s books was recoded with scores of two or less books to equal zero, and three or more to equal one thereby creating a binary variable of three plus books versus less than three.
Availability of playthings	Two or more playthings at home. Playthings include homemade toys, toys from shops, and household or outside objects.	Availability of playthings was recoded into a binary variable with two or more playthings, versus one or fewer.
Inadequate supervision	Child left alone or in the care of another child younger than ten for more than one hour in the last week.	A summary scored was created by recoding and computing two questions to create a binary alone versus not alone variable.
Violent physical discipline	Shook or spanked or hit or flicked ear or slapped or beat child up in past month.	A summary score was created by recoding and computing six questions to create a binary variable experiencing versus not experiencing violent physical discipline.
Psychological aggression	Shouting or yelling or screaming at, or calling child dumb, lazy or another derogatory name in the past month.	A summary score was created by recoding and computing six questions to create a binary variable for each, stipulating whether the child was or was not experiencing any of the forms of discipline.
Positive parenting	Explained why behaviour was wrong, gave child something else to do, and took away privileges in past month) • zero to two forms of positive parenting • three forms of positive parenting.	Three questions were recoded, summed and converted into a binary variable with the following classification: receiving zero to two forms, or receiving three forms of positive parenting.

Home based responsive caregiving and early learning factors were drawn from responses to questions about the home environment, based on UNICEF’s Family Care Indicators (FCI). The FCIs were validated against the Bayley Scales of Infant Development (BSID), with results showing that children in households with higher FCI scores (indicating more responsive caregiving and early learning) consistently scored higher on the BSID [55]. The indicators have demonstrated adequate test-retest reliability [56].

We extracted child factors including child age (as a continuous variable, and as a categorical variable by one year age groups), child sex (male, female).

We assessed family factors including: the number of children at home (one, two, three, four or more), maternal age (<25yo, 25–34yo, 35+), maternal education level (primary or lower, secondary or higher), marital status (married, not married), access to information (mother who at least once a week reads a newspaper or magazine listens to radio or watched TV, versus less than once a week), mother owns a mobile phone and mother feels safe walking alone in their neighbourhood after dark, and attitudes to domestic violence. Respondents were asked whether they think that husbands or partners are justified to hit or beat their wives or partners in a variety of situations, which aimed to capture the social justification of violence when a woman does not comply with certain expected gender norms.

We assessed socio-demographic factors including area of residence (urban, rural), division (Torba, Sanma, Penama, Malampa, Shefa, and Tafea), and household wealth. MICS surveys measure household wealth using a composite, asset-based index known as the principal components analysis (PCA) to assess cumulative living standards. This method was developed by World Bank to measure living standards using household survey data [57]. It ranks households on a relative scale, assigning them to one of five household wealth quintiles (poorest, poor, middle, better-off, richest). The ownership of consumer goods, dwelling characteristics, water, and sanitation are some examples of the information collected for the calculation of the wealth quintile [45].

**Data management and statistical analysis:** Data were managed and analysed using SPSS Version 29 [58]. MICS datasets are freely available to registered users. We sought and received permission from UNICEF to access the datasets by completing the online registration which included disclosure of the intended research purpose for the data. Raw Vanuatu MICS datasets of the information collected in the ‘individual woman’ and ‘children under 5’ (i.e., less than five years of age) surveys were accessed through the UNICEF data warehouse and downloaded to a password protected secure drive on the University’s secure server. Mother and child data sets were merged to create a master file of mother child pairs for children in the 24–59 month age range. Women who did not have children or children for whom data from the mother were not available, were not included. Missing value analysis was conducted in SPSS to review descriptive and tabular patterns of missingness across all variables. Missing value analysis indicated that all but one variable had complete data. This variable was created to determine the total number of sons and daughters at home, calculated by combining the sons at home variable and daughters at home variable. The sons and daughters at home variable showed 1.3% missingness (n = 15). Missing cases were scattered rather than clustered within a specific subgroup, indicating no discernible pattern. Given the minimal extent and likely to be missing at random, these were considered inconsequential and retained without imputation or case deletion.

Our data analyses comprised descriptive, regression and moderation analyses. First, descriptive data were calculated for each variable. Second, we examined the associations between explanatory factors and early childhood development index scores using a Complex Samples General Linear Model (CSGLM). The model included 22 predictors, comprising the predictor variables of interest (home caregiving and early learning variables), and child, household characteristics, safety, security and gender equality factors. The analyses were adjusted for sampling weights which were generated by MICS. To account for the clustered survey design, we created a single stratification variable by combining province and urban-rural residence. Household identifiers (H6 and HH7) were adjusted to form unique cluster codes, with HH7 multiplied by 10 before the two were combined. All analyses used the CSGLM, which incorporates stratification, clustering and sampling weights.

**Main model checks:** To assess for multicollinearity, we calculated Variation Inflation Factors (VIF) for all the variables in the models. A VIF of greater than five indicating likely collinearity would result in removing the variable from the model. To evaluate core regression assumptions, we examined both statistical tests and visual diagnostics of the model residuals. CSJLM provides raw residuals and predicted values but does not produce standardised residuals. Therefore, standardised residuals were created manually from the model generated residuals. The histogram of residuals showed an approximately bell shaped distribution, and the Q-Q plot demonstrated close alignment with the reference line, with only slight deviations of the tails. The residual versus fitted scatterplot displayed a random cloud of points around the zero with no discernible pattern, supporting the assumptions of linearity and homoscedasticity. The Shapiro-Wilk test indicated statistically significant deviation from normality (p = 0.047). However, given the large sample size this test is highly sensitive to minor departures. Overall, the diagnostics include no substantive violations of regression assumptions and support the adequacy of the model specification.

**Moderation analyses:** The third component of our statistical outputs were moderation analyses, conducted to assess the effects of five potential moderator variables (child age, child sex, urbanicity, maternal education and household wealth) on the associations between home caregiving and learning variables and child developmental outcomes. These variables are commonly used in moderation analyses because they represent key socio-economic, demographic, and environmental factors that can influence the relationship between child outcomes and early learning/ caregiving factors. For instance, developmental skills and responsiveness to stimulation increased rapidly between 24–59 months, so the effects of home learning activities often strengthen with age [1,2]. Boys and girls show different developmental trajectories and may receive different types of stimulation or discipline [3]. Urban rural differences in access to services, early learning opportunities, and resource can modify how strongly home environments influence development [35]. Maternal education shapes parenting knowledge, responsiveness, and the ability to provide stimulation often amplifying the benefits of home learning [59]. Wealth affects access to learning materials, nutrition, and caregiver time and socioeconomic contexts can strengthen or weaken the influence of home stimulation on development [1]. For ease of interpretation, we collapsed categorical variables into binary variables to create interaction terms. We conducted five CSGLM for each of the five moderator variables, with each model containing the 22 explanatory factors from the original regression model, plus the interaction terms.

**Ethics:** The Vanuatu Bureau of Statistics obtained approval for the survey protocol from the Vanuatu Health Research Ethics Committee in April 2023. It included a ‘protection protocol’ which outlines the potential risks during the life cycle of the survey and management strategies to mitigate these. Verbal consent was obtained for each respondent participating in the survey. All respondents were informed of the voluntary nature of participation, the confidentiality and anonymity of information, their right to refuse answering all or particular questions, as well as to stop the interview at any time.

## Results

**Fig 2:**
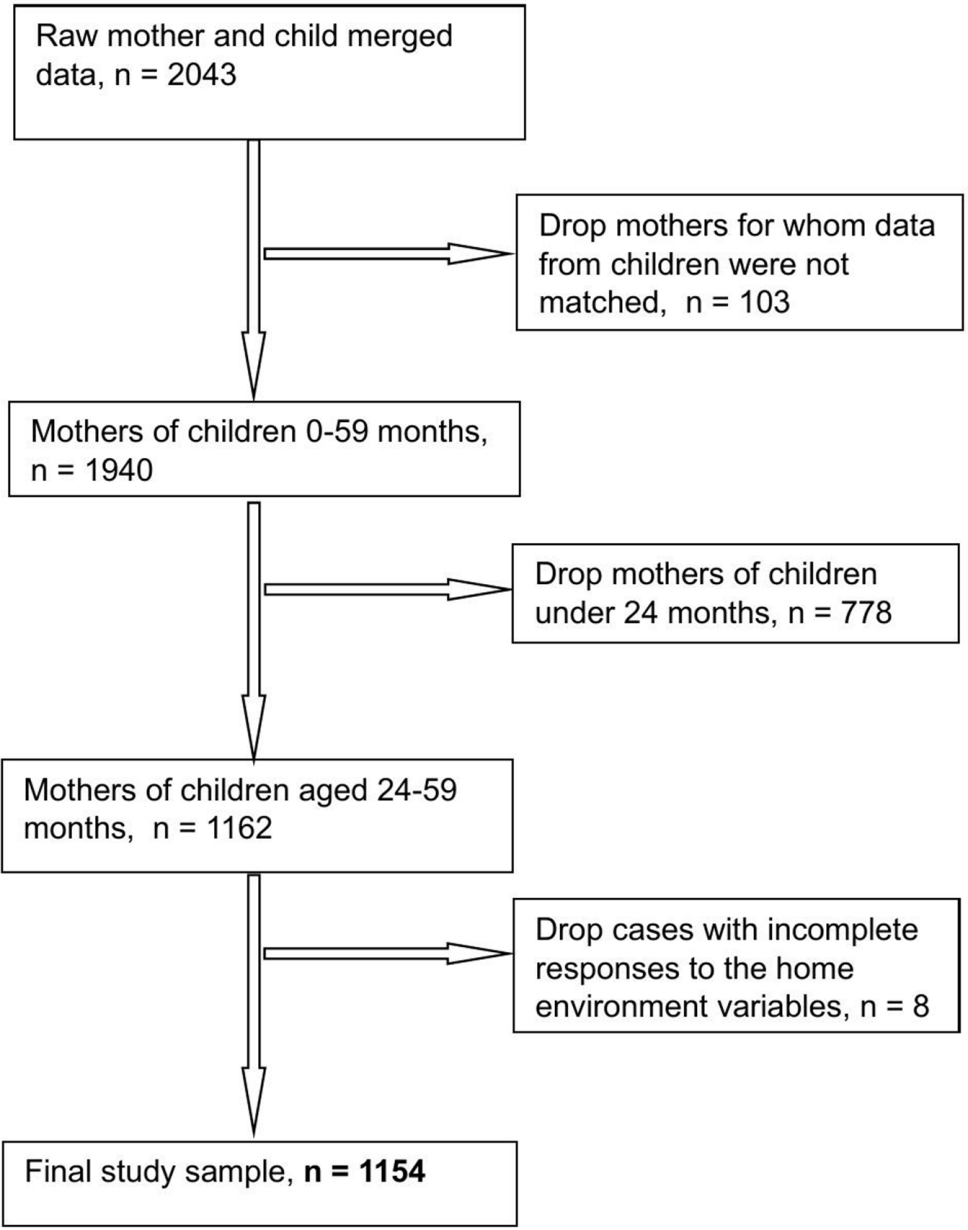
Study population flow chart.

A total of 2,043 mothers of children under five years were interviewed in the Vanuatu MICS, with a response rate of 98.1% within selected households. Of this sample, there were 1,162 mothers of children aged 24–59 months (Fig 2). We excluded eight cases due to incomplete responses to the home environment variables, resulting in a sample of 1,154.

### Socio-demographic, maternal and child characteristics

The two, three and four-year age groups comprised approximately one third each of the study sample (Table 2). The proportion of males was slightly higher than females. Almost four fifths of the respondents were from rural areas. Just over one quarter of the study sample was from Shefa, the most populated province in Vanuatu. The most remote region of Torba accounted for 5.7% of the study sample. Over half of the mothers were aged 25–34 years old, and around three fifths were educated to a secondary or higher level. The poorest wealth quintile accounted for one quarter of the sample.

**Table 2:** Socio-demographic, home-based responsive caregiving and early learning characteristics of sample (N = 1,154 children).

CHARACTERISTICS	n (%)
**Child age**	
24–35 months old	361 (31.3)
36–47 months old	407 (35.3)
48–59 months old	386 (33.4)
**Child sex**	
Female	559 (48.4)
Male	595 (51.6)
**Children at home**	
One	190 (16.6)
Two	347 (30.4)
Three	300 (26.3)
Four or more	302 (26.6)
**Area**	
Urban	254 (22.0)
Rural	900 (78.0)
**Division**	
Torba	66 (5.7)
Sanma	243 (21.1)
Penama	184 (15.9)
Malampa	112 (9.7)
Shefa	303 (26.3)
Tafea	246 (21.3)
**Maternal age**	
Less than 25 years old	163 (14.1)
25–34 years old	610 (52.9)
35 years and over	381 (33.0)
**Maternal education level**	
Primary or lower	471 (40.8)
Secondary or higher	683 (59.2)
**Marital status**	
Married	590 (51.1)
Not married	564 (48.9
**Household wealth quintile**	
Poorest	298 (25.8)
Poor	249 (21.6)
Middle	220 (19.1)
Better-off	229 (19.8)
Richest	158 (13.7)
**Communication**	
Mother who at least once a week read a newspaper or magazine, listened to the radio or watched TV	295 (25.6)
Less than once a week	859 (74.4)
**Own a mobile phone**	
Mother owns a phone	764 (66.2)
Mother doesn’t own a phone	390 (33.8)
**Attitudes to domestic violence (DV)**	
DV justified	744 (64.5)
DV not justified	410 (35.5)
**Neighbourhood safety**	
Mother feels safe in neighbourhood	674 (58.4)
Mother feels unsafe in neighbourhood	480 (41.6)
**Receiving adequate early stimulation and responsive care**	
By mother	904 (78.3)
By father	333 (28.9)
By someone other than mother or father	58 (5.0)
**Availability in the home of**	
children’s books (three or more books)	163 (14.1)
playthings (two or more playthings)	935 (81.0)
**Inadequate supervision**	400 (34.7)
**Violent physical discipline**	907 (78.6)
**Psychological aggression**	968 (83.9)
**Positive parenting**	
Two or fewer forms of positive parenting	536 (46.4)
Three forms of positive parenting	618 (53.6)

### Home-based early learning and responsive caregiving

Engaging in four or more of the following six activities in the past three days: reading books to, telling stories to, singing songs with, taking the child outside, playing with, naming or counting, were undertaken by 78.3% of mothers and 28.9% of fathers and 5% by someone other than the mother or father (Table 2). In total, 14% of households had at least three books in the home, while 81% of households had at least two playthings at home. Approximately one third of children were left alone or in the care of another child aged younger than ten years for more than one hour in the previous week. Widespread use of discipline was reported; 78.6% of children experienced physical discipline, while 83.9% experienced psychological aggression. In the past month, two or fewer forms of positive parenting were experienced by 46.6% of children, while 53.6% of children receiving three forms of positive parenting.

### Early childhood development

In the overall study sample, 68.2% of children were developmentally on track, meaning they had achieved the ECDI2030 minimum milestones set by UNICEF (Table 3). However, this proportion decreased with increasing age: 72% of children aged 24–35 months were on track, compared to 69.8% of those aged 36–47 months, and 63% for children aged 48–59 months. The mean ECDI2030 scores increased with increasing age.

**Table 3:** Distribution of ECDI2030 scores and ECDI2030 milestone achievement (N = 1154 children).

Age Groups	ECDI2030 score Mean (SD)	ECDI2030 milestone achieved n (%)
24 to 35 months	9.69 (4.01)	260 (72.0)
36 to 47 months	12.86 (3.67)	284 (69.8)
48 to 59 months	15.13 (3.39)	243 (63.0)
**TOTAL**	12.63 (4.29)	787 (68.2)

### Main model assumption and collinearity checks

Variation Inflation Factors were checked for all the variables in the model. None had a score of greater than five indicating no collinearity in the variables in the model. The histogram of residuals showed an approximately bell shaped distribution, and the Q-Q plot demonstrated close alignment with the reference line, with only slight deviations of the tails. The residual versus fitted scatterplot displayed a random cloud of points around the zero with no discernible pattern, supporting the assumptions of linearity and homoscedasticity. The Shapiro-Wilk test indicated statistically significant deviation from normality (p = 0.047). However, given the large sample size this test is highly sensitive to minor departures. Overall, the diagnostics include no substantive violations of regression assumptions and support the adequacy of the model specification.

### Associations between explanatory factors and mean early childhood development score

We assessed the associations between indicators of early childhood development and 22 explanatory factors in the linear regression model presented in Table 4. All variables had variation inflation factors of less than three, suggesting no collinearity. Null findings were reported for ten of the twenty-two factors investigated.

**Table 4:** Associations between potential factors and ECDI2030 score among children 24-59 months (N = 1154).

Variable	Regression coefficient^a^	p-value	95% CI
**Sex**			
Male	Reference		
Female	0.27	0.165	-0.11 to 0.64
**Children at home**			
One	Reference		
Two	0.41	0.166	-0.17 to 0.99
Three	-0.09	0.818	-0.76 to 0.6
Four or more	0.15	0.645	-0.51 to 0.82
**Mother age**			
Less than 25yo	Reference		
25 to 34yo	-0.141	0.658	-0.77 to 0.49
35 plus	-0.23	0.513	-0.93 to 0.47
**Maternal education level**			
Secondary or higher	Reference		
Primary or lower	-0.13	0.590	-0.61 to 0.35
**Marital status**			
Married	Reference		
Not married	-0.24	0.250	-0.65 to 0.17
**Urbanicity**			
Urban	Reference		
Rural	-0.85	0.012*	-1.52 to - 0.19
**Division**			
Shefa	Reference		
Torba	0.652	0.354	-7.32 to 2.04
Sanma	-1.40	< 0.001*	-2.15 to - 0.64
Penama	1.97	< 0.001*	1.20 to 2.73
Malampa	-0.184	0.670	-1.04 to 0.67
Tafea	0.47	0.253	-0.34 to 1.28
**Household wealth quintile**			
Richest	Reference		
Better-off	0.83	0.012*	0.19 to 1.47
Middle	0.16	0.721	-0.71 to 1.02
Poor	0.22	0.654	-0.75 to 1.19
Poorest	-0.60	0.257	-1.65 to 0.44
**Communication**			
Mother who at least once a week read a newspaper or magazine, listened to the radio or watched TV	Reference		
Less than once a week	0.35	0.125	-0.10 to 0.81
**Own a mobile phone**			
Mother owns a phone	Reference		
Mother doesn’t own a phone	.09	0.741	-0.45 to 0.63
**Violent discipline**			
Not used on child	Reference		
Used on child	0.25	0.367	-0.30 to 0.80
**Psychological aggression**			
Not used on child	Reference		
Used on child	-0.13	0.693	-0.76 to 0.50
**Positive parenting**			
Receiving three forms	Reference		
Receiving two or fewer forms of positive parenting	-1.22	<0.001*	-1.79 to -0.95
**Attitudes to domestic violence (DV)**			
DV justified	Reference		
DV not justified	-0.67	0.006*	-1.14 to -020
**Neighbourhood safety**			
Mother feels safe in neighbourhood	Reference		
Mother feels unsafe in neighbourhood	0.13	0.536	-0.29 to 0.55
**Mother stimulation and responsive care**			
Adequate stimulation and responsive care from mother	Reference		
Inadequate stimulation and responsive care from mother	-0.61	0.027*	-1.16 to -0.07
**Father stimulation and responsive care**			
Adequate stimulation and responsive care from father	Reference		
Inadequate stimulation and responsive care from father	-0.53	0.031*	-1.01 to -0.05
**Stimulation and responsive care by someone other than father or mother**			
Adequate stimulation and responsive care from other	Reference		
Inadequate stimulation and responsive care from other	-1.93	< 0.001*	-2.73 to -1.12
**Availability of books**			
Three or more books	Reference		
Less than three books	-1.77	< 0.001*	-2.35 to -1.19
**Availability of playthings**			
Two or more playthings	Reference		
One or less playthings	-0.84	0.004*	-1.41 to -0.26
**Supervision**			
Adequate supervision	Reference		
Inadequate supervision - left alone one hour+ or with another child under 10yo	0.57	0.020*	0.09 to 1.04
**Child age (in months)**	0.21	<0.001*	0.19 to 0.23

^a^Estimated by a multiple linear regression model.

Eight home responsive caregiving and early learning factors were significantly associated with child development scores. Children receiving two or fewer forms of positive parenting had significantly lower scores than those receiving at least three forms. Development scores were significantly lower for children receiving inadequate versus adequate stimulation and responsive care from the mother, father, or nonparent caregiver. Children with fewer than three books at home had significantly lower scores compared with children who had three or more books. Similarly, children with one or fewer playthings at home had significantly lower scores than children with two or more playthings at home. Children experiencing inadequate supervision had significantly higher developmental scores compared with children with adequate supervision.

Five socio-demographic, mother and child factors were significantly associated with child development scores. Children from rural areas had significantly lower developmental scores compared with children from urban areas. Children in the Sanma region had significantly lower development scores than those in Shefa, whereas children in Penama region had significantly higher scores. Mean developmental scores were significantly higher for older children. Developmental scores were higher for children whose families justified domestic violence, compared with those who did not.

### The moderating role of socio-demographic factors on home-based early learning and responsive caregiving and child development

Moderation analyses explored how the associations between eight home-based early learning and responsive caregiving factors and child development change depending on socio-economic factors. We found significant findings among four of the five moderator variables assessed in the moderation analyses (Table 5). The association between supervision and child development was stronger for older than younger children. Having books at home and child development was stronger for boys than girls. The association between stimulation and responsive caregiving by someone other than the parent and child development was stronger for boys than girls. Positive parenting and child development was stronger for girls than boys. Positive parenting and child development was stronger for urban than rural children. Positive parenting and child development was stronger for children whose mothers has less education. Higher maternal education was associated with lower psychological discipline, indicating that children of more educated mothers were more likely to avoid psychological discipline. No statistically significant associations were observed for wealth across any of the outcomes.

**Table 5:** Results of moderation analyses testing the effects of five potential moderator variables on the relationship between home-based early learning and responsive caregiving practices on ECD outcomes.

	Child age in months	Child sex (Male = 1; female = 0)	Urbanicity (Urban = 1; Rural = 0)	Maternal education (Secondary+ = 1; Primary school = 0)	Wealth (reference = richest) (Better off = 1, worse off = 0
	**B, 95%CI**				
**Mother stimulation and responsive care** 0 = insufficient 1 = adequate	-0.02 (-0.07 to 0.03)	0.51 (-0.53 to 1.55)	0.32 (-0.86 to 1.49)	-0.11 (-0.86 to 0.64)	-0.30 (-1.41 to 0.81)
**Father stimulation and responsive care** 0 = insufficient 1 = adequate	0.02 (-0.03 to 0.06)	-0.17 (-1.08 to 0.73)	-0.14 (-1.32 to 1.05)	-0.55 (-1.35 to 0.25)	0.17 (-0.80 to 1.14)
**Stimulation and responsive care from someone other than parent** 0 = insufficient 1 = adequate	-0.06 (-0.14 to 0.01)	1.62 (-0.30 to 2.95) *	0.37 (-1.19 to 1.93)	0.34 (-0.85 to 1.53)	-1.04 (-3.07 to 0.98)
**Availability of books** 0 = Fewer than three 1 = Three or more	-0.01 (-0.05 to 0.07)	1.24 (0.06 to 2.43) *	1.17 (-0.05 to 2.38)	0.79 (-0.19 to 1.76)	-0.76 (-2.06 to 0.55)
**Availability of playthings** 0 = One or less 1 = Two or more	-0.03 (-0.07 to 0.01)	0.59 (-0.35 to 1.54)	-0.47 (-2.64 to 1.70)	-0.11 (-0.91 to 0.69)	-0.08 (-1.25 to 1.09)
**Adequate supervision** 0 = Unsupervised 1 = Supervised.	**0.04** (0.002 to 0.08) *	-0.36 (-1.18 to 0.47)	-0.79 (-1.98 to 0.41)	0.31 (-0.42 to 1.03)	0.48 (- 0.52 to 1.48)
**Positive parenting** 0 = Two or fewer forms; 1 = Three forms.	-0.02 (-0.06 to 0.02)	-0.99 (-1.78 to -0.19) *	1.44 (0.32 to 2.56) *	-1.13 (-1.79 to -0.46) *	-0.64 (-1.48 to 0.21)
**Psychological discipline** 0 = Experiencing 1 = Not experiencing	-0.03 (-0.09 to 0.04)	-0.95 (-2.27 to 0.37)	-1.21 (-2.58 to 0.16)	0.98 (0.13 to 1.82) *	0.93 (-0.51 to 2.37)
**Violent discipline** 0 = Violent 1 = Not violent.	0.04 (-0.01 to 0.08)	0.36 (-0.63 to 1.35)	0.85 (-0.29 to 2.00)	-0.22 (-1.16 to 0.72)	-0.59 (-1.75 to 0.57)

Note: * p < 0.05; Cell data: regression coefficient [95% CI] of the interaction term adding to the multiple regression model presented in Table 4.

## Discussion

In this secondary analysis of data from the 2023 Vanuatu MICS, we examined the associations between home-based early learning and responsive caregiving, socio-demographic factors, and the development of children aged 24–59 months. When adjusted for covariates, we found significantly higher development scores for children with three or more books, two or more playthings, receiving three forms of positive parenting, experiencing adequate stimulation and responsive care from the mother, father, and nonparent caregiver. Among socio-demographic factors we found that mean developmental scores were significantly higher for older versus younger children, urban versus rural children, and significantly different outcomes between regions in Vanuatu. Developmental scores were also significantly higher for children whose families justified domestic violence, compared with those who did not, and children experiencing inadequate versus adequate supervision. Null findings were reported for ten of the twenty-two factors investigated.

Our findings of significant associations between higher development and three or more books at home highlight the importance of early learning resources in supporting child development. Although the associations were strong, only 14% of children in this sample had three or more books at home, below the 23.5% average reported in a cross-sectional study of 68 LMICs [60]. The low availability of children’s books in Vanuatu may be due to limited access in remote areas, economic constraints, a lack of books in local languages, as well as strong oral storytelling traditions [61]. Shared book reading between the caregiver and child supports early development by enhancing language acquisition, cognitive skills, emotional bonding, and brain development through structured, nurturing interactions [7,16]. Our results align with previous research showing that the presence of books at home is positively associated with child development [ 28,31,60,62–64]. A meta-analysis across 43 LMICs found links between the home environment, including book availability, and children’s language and literacy skills [65]. Similarly, a study of 35 LMICs showed that having children’s books at home nearly doubled the likelihood of children being on track for numeracy and literacy, with the greatest benefits in lower income countries [28].

We found a significant association between having two or more playthings at home and higher development scores, highlighting the importance of play in early development. Notably, 81% of children in our study had two or more playthings; substantially higher than the 54% reported in a multi-country study which included items like: household objects or objects found outside (sticks, rocks, animals, shells, leaves) or homemade or store bought toys [60]. The Nurturing Care Framework highlights early stimulation and play as foundational for healthy development, regardless of the play materials used [7]. In resource constrained settings, play with homemade items can promote development through cognitive, motor, language, and social development, while reinforcing cultural relevance and caregiver engagement [66]. In Vanuatu, use of natural and household materials for play and learning is common, often substituting for books [61].

Engagement by the caregiver in stimulating early learning activities such as playing and reading is strongly associated with better child development across multiple studies [23,26,34,67–69]. Consistent with prior research, children tend to experience higher levels of cognitive stimulation from their mothers than their fathers [31,69,70]. In Vanuatu, levels of stimulation from mothers (78.3%) and fathers (28.9%) were substantially higher than other studies. A cross-sectional study of 47 LMICs reported that on average, 34.7% of young children received four or more stimulating activities from their mothers compared with only 14.1% from their fathers [70]. Similarly, a study of parent stimulation in 62 LMICs showed that 39.8% of mothers, and 11.9% of fathers provided high levels of stimulation at home [69].

We found significant associations between higher developmental scores and adequate stimulation and responsive care from mothers, fathers and from non-parental caregivers. In Vanuatu, particularly in rural areas, cultural norms often emphasise children learning through observation, participation, and assisting with household tasks alongside parents [71]. However, the survey’s paternal involvement items may not have fully captured the ways fathers typically engage with their children, for instance through shared work or informal teaching, resulting in a potentially incomplete picture of their contribution. Furthermore, the questionnaire module did not cover activities that children engage in with adults that are not members of the household, even when these occur frequently, limiting a comprehensive understanding of children’s caregiving environments [45]. Alloparenting, which is defined as the provision of care by individuals other than the child’s biological parents, is a key aspect of child rearing and is common practice in Pacific Island countries [72]. The quality and quantity of this alloparental care have been shown to influence outcomes in children [73].

Higher child development scores were strongly associated with receiving three forms of positive parenting, in line with prior research [21,22,31]. Positive parenting strategies involve helping the child to understand why a behaviour is inappropriate, redirecting them to an alternate activity, and when necessary, removing certain privileges [45]. Frequent and high-quality interactions between the caregiver and child are positively associated with better developmental outcomes [23].

High levels of physical (78.6%) and psychological (83.9%) discipline among children were reported, though these were not significantly associated with lower development scores in regression analyses. These levels are notably higher than those reported in a secondary analysis of 107,000 children aged 24–59 months across 49 LMICs, which found average prevalence of 62.5% for physical and 65.4% for psychological discipline [74]. Several factors may explain the lack of significant associations. Variations in the timing and duration of exposure can influence whether effects are immediately observable. Cultural norms may lead to underreporting of harsh discipline. Where practices are pervasive, there is no comparison group in the setting. Furthermore, a child exposed to violence may still show typical development due to protective factors such as strong attachment to a non-abusive caregiver [75]. Nevertheless, prolonged, or severe exposure to harsh discipline can trigger toxic stress, which is known to disrupt early brain development and long-term health outcomes [1].

Our study found that children from families who justified domestic violence had significantly higher developmental scores than those who did not. While such attitudes do not directly endorse or predict violence, they may reflect broader social tolerance for gender-based violence rooted in entrenched gender norms and hierarchies [76,77]. These patterns align with broader gender norms in Vanuatu, where high social acceptance of physical discipline and intimate partner violence reflects entrenched hierarchies within family life [49]. Such norms may help contextualise why attitudes justifying violence appear in our data and how they might intersect with caregiver dynamics that influence children’s developmental experiences. However, relationship quality, including emotional support, communication, and co-parenting practices may confound these findings since it influences both attitudes towards violence and child development [78]. Given the cross-sectional study design, these results indicate an association, not causation. Our findings contrast with a review of exposure to domestic violence which found that children tended to experience a range of negative developmental outcomes including being at increased risk of behavioural and emotional problems [79].

Children who were left alone or in the care of another child younger than ten for more than one hour in the previous week had higher developmental scores than children not left alone. This counterintuitive finding may reflect limitations in the brevity of the supervision measure, which may not fully capture the quality or context to supervision. Reverse causation is also possible: caregivers may be more willing to leave children who are perceived as developmentally competent [80,81]. In rural areas children moving independently may reflect culturally normative caregiving practices rather than neglect, as suggested by interaction analysis. This finding may reflect exploratory play, where less supervision fosters imagination and problem solving which are key drivers of early development [82]. However, this should not be interpreted as an endorsement of leaving children unsupervised. In contexts where caregivers must work to support their families, leaving children alone may be a necessity rather than a choice. These findings highlight the need for nuanced interpretation in the context of socioeconomic realities.

Our finding that 68.2% of children were developmentally on track was lower than that reported for other countries in the East Asia and Pacific region that have released ECDI2030 data. On track percentages for 24–59 month old children were 83.0% for Fiji [83], 78.2% for Vietnam [84], and 77.8% for Thailand [85]. Differences in children’s developmental outcomes across countries may reflect variations in the national income levels, poverty rates, and the scale and quality of investment in early childhood development. While all four countries have made commitments to child development, the scope of their investments and the influence of culture and conceptual factors differ [86].

Our findings of higher mean developmental scores for older versus younger children is consistent with another study conducted in Vanuatu and the East Asia-Pacific region [35]. Evidence from LMIC settings show that developmental trajectories do not always increase steadily with age. McCoy et al. (2016) highlight that cumulative exposure to poverty and undernutrition can result in older preschoolers showing higher predicted rates of developmental risk than younger children [2]. Similarly, a systematic review measuring ECD in LMICs showed that several tools show age-related dips or plateauing in older children, particularly in language and social-emotional domains [87].

However, when applying UNICEF’s milestone cut offs across the two, three, and four year-old age group, the four year olds were the least likely to be ‘on track’ for development in our sample. This pattern aligns with findings from validation studies confirming the suitability of the ECDI2030 for monitoring development in children aged 24–59 months despite the age group differences [53]. Validation studies conducted in Mexico and Palestine to determine the age-specific milestone cut-scores showed that a higher percentage of 48 month olds were not developmentally on track (22%), compared to 24 month olds (14%) [88]. As more countries implement the MICS survey through to 2026, UNICEF acknowledges that emerging data will inform potential revisions to the cut-scores [54]. These will help to clarify whether the observed differences reflect actual developmental faltering or limitations in the sensitivity of the current scoring thresholds. Currently, only 39.4% of three and four year old children in Vanuatu participate in centre based education, potentially hindering development. [45] Additionally, the low provision of home stimulation and early learning may be a factor impacting development.

In line with other studies, we found that urban versus rural children had higher developmental scores [35,89–91]. Possible explanations include more stimulating environments in urban areas [91,92], and the higher likelihood of wealthier households in urban areas contributing to better development [89,90].

Our results of significantly different child development scores between regions, highlights the variations in poverty, cultural and community practices, and provincial differences in post-disaster recovery efforts in Vanuatu [45]. Despite being more rural, Penama was associated with higher child development than the province of Shefa, home to Vanuatu’s capital, Port Villa. This may be due to strong community-based caregiving, and child-focused development programs following the 2017 and 2019 Ambae volcanic eruptions [93]. Sanma’s lower scores compared with Shefa could reflect the higher poverty rates and the lingering disruption to health and education services since the province was severely affected by Cyclone Harold in 2020 [45].

Moderation analyses showed that the benefits of home based early learning and responsive caregiving varied by socio-demographic group. Having books at home showed a stronger association for boys, reflecting that book access represents a greater departure from prevailing norms for boys in Vanuatu, thereby delivering a larger developmental gain. School-based analyses reveal gendered expectations in learning environments, with boys and girls often encouraged to adopt different roles and behaviours in the classroom, including differential support for literacy related activities [94]. Broader gender equality assessments corroborate the persistence of masculine norms, often characterised by physicality, toughness, and reduced emphasis on academic behaviours [95]. These gendered expectations continue to shape children’s opportunities and everyday behaviours in Vanuatu and within this context, book access may amplify the developmental benefits among boys.

Supervision was more strongly linked to the development in older children, possibly reflecting the value of guided autonomy at later stages. The stronger associations in older children align with evidence that responsiveness to stimulation increases rapidly between 24–59 months, giving home interactions greater influence as children age [2]. Positive parenting was more strongly associated with the development of girls, urban children, and those whose mothers had less education. These patterns may reflect gendered caregiving practises, more emphasis on parenting skills in urban settings, and a compensatory effect where formal maternal education is limited. Differences by gender and urban rural residence reflect broader findings that boys and girls receive different types of stimulation and that access to services and early learning opportunities varies across settings [3,35]. Although the literature is limited on the moderating role of socio-demographic factors on the relationship between home-based early learning and responsive caregiving and ECD, some studies confirm the influence of maternal education on child outcomes [96,97].

### Strengths and limitations

Strengths of this secondary analysis include: the 2023 Vanuatu MICS was nationally representative, the survey was administered by trained enumerators, and the questionnaire was customised and translated from English into Bislama and French, and pre-tested in both urban and rural areas.

Nevertheless, we acknowledge some limitations. Moderation analyses of secondary data are useful for exploring complex relationships but remain exploratory. Marginal affects should be interpreted cautiously due to the increased risk of type 1 error from multiple testing. There are some limitations regarding the source data. First, the MICS survey provides broad, population-level insights but lacks the granularity of individual developmental assessments. Second, the survey omits activities involving non-household adults, who often play key caregiving roles in Vanuatu communities [45]. This might overlook important aspects of a young child’s experiences. Third, the child development and caregiving data were based on caregiver -reports, which are subject to biases. Parent reporting may increase the likelihood of social desirability bias where answers are overstated to appear more acceptable, and by recall bias where past events are remembered inaccurately [98]. Fourth, the responsive caregiving measure included only six general activities over the past three days, without assessing quality or frequency of interactions. Fifth, the study’s cross sectional nature limits causal inference. While associations can be identified, they cannot be interpreted as cause-effect relationships.

### Implications for research, policy and practice

This study provides local data from the perspective of caregivers, which is essential for shaping early childhood development policies that reflect the cultural and socioeconomic realities of Vanuatu. Findings from our secondary analyses highlight potential policy priority areas including promoting access to books and toys, investing in parenting education to strengthen home-based support for child development, engaging fathers in caregiving through gender-informed approaches, and tailoring programs to regional and rural contexts. More broadly, poverty reduction strategies could benefit children in Vanuatu. Suggested future research includes investigating the role of father involvement and examining the unexpected associations between justification of domestic violence, supervision, and child development.

### Conclusion

Using data from the 2023 Vanuatu MICS, this study quantified the associations between home-based early learning and responsive caregiving, socio-demographic factors, and the development of children aged 24–59 months. Adjusted analysis showed higher developmental scores among children with greater access to learning resources, more positive parenting and responsive care, older age, urban residence and, unexpectedly among those with inadequate supervision or whose families justified domestic violence. Findings point to policy priorities such as expanding access to books and toys, strengthening caregiver education, engaging fathers, and tailoring support to regional and rural contexts. Addressing geographical disparities, and furthering research surrounding the home caregiving and early learning environment are important next steps for advancing child development in Vanuatu.
